# Allura Red AC is a xenobiotic. Is it also a carcinogen?

**DOI:** 10.1093/carcin/bgae057

**Published:** 2024-08-12

**Authors:** Lorne J Hofseth, James R Hebert, Elizabeth Angela Murphy, Erica Trauner, Athul Vikas, Quinn Harris, Alexander A Chumanevich

**Affiliations:** Department of Drug Discovery and Biomedical Sciences, College of Pharmacy, University of South Carolina, Columbia, SC, 29208, United States; Cancer Prevention and Control Program, University of South Carolina, Columbia, SC, 29208, United States; Department of Epidemiology and Biostatistics, University of South Carolina, Columbia, SC, 29208, United States; Department of Pathology, Microbiology & Immunology, School of Medicine, University of South Carolina, Columbia, SC, 29208, United States; Department of Drug Discovery and Biomedical Sciences, College of Pharmacy, University of South Carolina, Columbia, SC, 29208, United States; Department of Drug Discovery and Biomedical Sciences, College of Pharmacy, University of South Carolina, Columbia, SC, 29208, United States; Department of Drug Discovery and Biomedical Sciences, College of Pharmacy, University of South Carolina, Columbia, SC, 29208, United States; Department of Drug Discovery and Biomedical Sciences, College of Pharmacy, University of South Carolina, Columbia, SC, 29208, United States

**Keywords:** Allura Red AC, DNA damage, colorectal cancer, microbiome, inflammation

## Abstract

Merriam-Webster and Oxford define a xenobiotic as any substance foreign to living systems. Allura Red AC (a.k.a., E129; FD&C Red No. 40), a synthetic food dye extensively used in manufacturing ultra-processed foods and therefore highly prevalent in our food supply, falls under this category. The surge in synthetic food dye consumption during the 70s and 80s was followed by an epidemic of metabolic diseases and the emergence of early-onset colorectal cancer in the 1990s. This temporal association raises significant concerns, particularly given the widespread inclusion of synthetic food dyes in ultra-processed products, notably those marketed toward children. Given its interactions with key contributors to colorectal carcinogenesis such as inflammatory mediators, the microbiome, and DNA damage, there is growing interest in understanding Allura Red AC’s potential impact on colon health as a putative carcinogen. This review discusses the history of Allura Red AC, current research on its effects on the colon and rectum, potential mechanisms underlying its impact on colon health, and provides future considerations. Indeed, although no governing agencies classify Allura Red AC as a carcinogen, its interaction with key guardians of carcinogenesis makes it suspect and worthy of further molecular investigation. The goal of this review is to inspire research into the impact of synthetic food dyes on colon health.

## Teaser

Allura Red AC is ubiquitous in ultra-processed foods. Despite lacking carcinogen classification, its impact on colon health demands further investigation. This review summarizes our knowledge of the interaction of Allura Red AC with DNA, inflammatory players, and the microbiome.

## Graphical Abstract

**Graphical Abstract:**
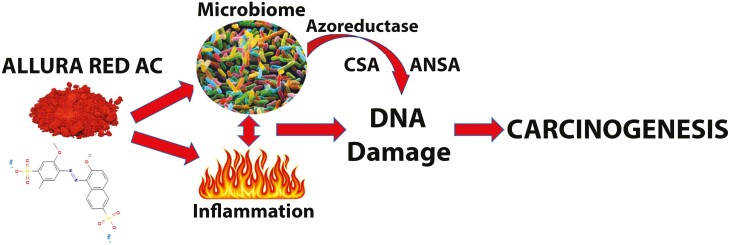

## Introduction and history of Allura Red AC in the USA

A xenobiotic is defined by the Oxford dictionary as “relating to or denoting a substance, typically a synthetic chemical, that is foreign to the body or to an ecological system.” Merriam-Webster describes it as “a chemical compound that is foreign to a living organism.” Food dyes, often synthesized from petroleum or crude oil [1–5], are considered xenobiotics and are approved globally to enhance the color of processed foods. However, the question remains: Are they carcinogens? According to the Oxford dictionary, a carcinogen is “any agent that (directly or indirectly) causes cancer or induces malignant transformation of cells,” while Merriam-Webster defines it as “a substance or agent causing cancer.” Although there is no direct evidence that Allura Red AC alone causes cancer, it is important to note that experiments specifically designed to investigate Allura Red AC’s potential to accelerate the development of colorectal and other cancers have not yet been conducted. Nonetheless, this review will present evidence that Allura Red AC impacts the carcinogenic process.

The widespread use of synthetic food dyes, including Allura Red AC, since the 1970s correlates with a rise in reported allergies and immune-reactive disorders [6, 7] (short-term impact) and an increase in chronic diseases such as type 2 diabetes mellitus, metabolic dysfunction-associated steatotic liver disease, and early-onset colorectal cancer (EOCRC) [8–14] (long-term impact). Over the past 50 years, the use of synthetic dyes in foods has surged [5], coinciding with a concerning rise in childhood behavioral issues [15] and chronic health conditions appearing at earlier ages [16, 17]. An estimated 8% of children with attention deficit hyperactivity disorder (ADHD) may have symptoms related to synthetic food colors [18]. These dyes, readily absorbed through food, cosmetics, and pharmaceuticals, present challenges to the immune system due to their small molecular size. They have the potential to trigger inflammatory responses, compromise intestinal permeability, provoke cross-reactivities, instigate autoimmune reactions, and contribute to neurobehavioral disorders. The long-term effects of chronic consumption on chronic diseases are still not fully understood. Despite these concerns, regulatory bodies like the Food and Drug Administration (FDA) in the USA and the European Food Safety Authority (EFSA) continue to permit high concentrations of synthetic colorants in everyday consumables.

The rich colors of naturally anti-inflammatory foods are directly related to the phenolic compounds that are associated with color [19, 20]. In ancient times, drugs and cosmetics used naturally derived color additives sourced from botanicals and minerals. Paprika, turmeric, saffron, as well as iron and copper sulfate stand as enduring examples of these ancient coloring agents. Across civilizations, from the Egyptians to the Greeks, the infusion of color into cosmetics and even beverages like wine dated back to at least 300 BC [21]. The landscape of color additives transformed dramatically with the serendipitous discovery by William Henry Perkin in 1856 of the first synthetic organic dye, known as mauveine—a purple dye. This breakthrough introduced a new era, where many more synthetic dyes soon followed. These innovations rapidly found their way into coloring foods, drugs, and cosmetics. Originating from coal processing byproducts, they earned the moniker “coal-tar colors” [22].

The rising popularity of synthetic color additives prompted the US government to initiate regulatory oversight. As early as the 1880s, the United States Department of Agriculture initiated research on the safety of color additives in food. By 1900, numerous products in the USA, including foods, drugs, and cosmetics, featured artificial coloring. However, safety concerns emerged due to reports of hazardous substances such as lead, arsenic, and mercury being covertly added to these products [23]. In response to concerns, the United States Congress passed the Food and Drugs Act in 1906, signaling a move toward safeguarding public health. This legislation aimed to curb the use of deleterious color additives, particularly in confectionery, and to uncover food defects. Subsequent developments over the next decades led to the establishment of the FDA in 1927, providing more robust regulatory oversight. The Federal Food, Drug, and Cosmetic Act of 1938 expanded this oversight to include cosmetics and medical devices. This legislative overhaul introduced strict standards for the safety and effectiveness of color additives, requiring premarket approval and certification procedures. Particularly noteworthy were the Food Additive Amendment of 1958 and the Color Additive Amendment of 1960, which emphasized increased scrutiny and implemented safeguards against potential carcinogens, notably through the inclusion of the “Delaney Clause” in 1958. This clause prohibits the listing of any color additive found to induce cancer when ingested by humans or animals [24]. Allura Red AC is not one of them yet.

The authorized food dyes in the European Union (EU) and the USA are discussed in other sources [25]. Presently, in the EU 39 colors are authorized as color additives for use in foods. In the USA, there are 36 approved food dye additives of which nine of artificial or synthetic origin, are subject to certification. These nine are FD&C Blue No. 1 (Brilliant Blue FCF; E133), FD&C Blue No. 2 (Indigo Carmine; E132), FD&C Green No. 3 (Fast Green FCF; E143), FD&C Red No. 3 (Erythrosine; E127), FD&C Red No. 40 (Allura Red AC; E129), FD&C Yellow No. 5 (Tartrazine; E102), FD&C Yellow No. 6 (Sunset Yellow FCF; E110), Citrus Red No. 2 (Amaranth; E121), and Orange B [26]. The latter five (Allura Red AC, Tartrazine, Sunset Yellow, Citrus Red No. 2, and Orange B) are azo dyes, the metabolic consequences of which will be described below. Notably, three of the nine synthetic food dyes approved in the USA are not permitted in the EU, namely Orange B, Citrus Red No. 2, and FD&C Green No. 3 [25].

Allura Red AC was approved for use in food by the FDA in 1971 [27], the subsequent widespread use of Allura Red AC in the food industry grew rapidly. Its approval by regulatory authorities in 1971 was based on initial unpublished safety assessments that focused primarily on acute toxicity studies in laboratory animals and observations of short-term effects in humans [28]. However, the long-term health implications of regular consumption of Allura Red AC and other synthetic food dyes remain understudied. Alarmingly, 20 years later, we started seeing a rise in EOCRC [29] and other chronic diseases [30]. Is it the Allura Red AC, other synthetic food dyes, or other ingredients in ultra-processed foods we consume? We posit Allura Red AC is a contributing factor.

Despite emerging concerns about the potential health risks associated with Allura Red AC, regulatory agencies continue to permit their use in food products, due to their perceived safety. However, studies conducted in recent years have raised questions about their safety. The Southampton Study, formally known as the “Southampton Food Standards Agency Study of Food Additives and Hyperactivity,” was a ground-breaking research project conducted by scientists at the University of Southampton in the UK. Published in 2007 [7], the study gained significant attention for its findings suggesting that the consumption of Sunset Yellow (E110), Quinoline Yellow (E104), Carmoisine (E122), Tartrazine (E102), Ponceau 4R (E124), and Allura Red AC (E129) was associated with increased hyperactivity in children [7]. This research contributed to discussions about the regulation of food additives and their potential impact on behavior, particularly in children with hyperactive disorders like ADHD. It also led to policy change in 2008, when the European Parliament decreed those foods containing synthetic food dyes, including Allura Red AC, must be labeled with the name or the E-number information followed by the warning “may have an adverse effect on activity and attention in children” [21]. Although subsequent studies and reviews have produced mixed results, there is a general acceptability that synthetic food dyes have a neurological and immunological impact. Consistent with this, Noorafshan *et al.* [31] investigated the neurotoxicity of low-dose (7 mg/kg/day) and high-dose (70 mg/kg/day) Allura Red AC on rats’ medial prefrontal cortex, with results showing memory impairment and structural changes in the medial prefrontal cortex in both low and high Allura Red AC-exposed groups. Alarmingly, the total number of the glial cells was reduced significantly with low-dose Allura Red AC. Both doses of Allura Red AC caused an increase in working and reference memory errors. Allura Red AC also induces β-lactoglobulin protein fibrillation, further supporting the notion that this xenobiotic may contribute to neurodegenerative disorders [32]. This review examines the potential effects of chronic Allura Red AC exposure on the colon. Despite limited knowledge, it highlights the complex relationship between technological advances, regulations, industry influence, and scientific exploration. Ongoing research is crucial to safeguard consumer health in our processed food landscape.

## Allura Red AC exposure has grown over the past 50 years

Science has borne out that consuming an ultra-processed diet poses a significant health risk, contributing to multiple chronic conditions such as obesity, type 2 diabetes, cardiovascular disease, and cancer [33, 34]. These diseases collectively account for approximately 80% of all noncommunicable diseases globally [35, 36]. In the USA, over 73% of the food supply is ultra-processed, incorporating additives not intended for traditional food preparation, with lower-income populations exhibiting particularly high consumption rates [37, 38]. Alarmingly, the consumption of ultra-processed foods, often containing synthetic food dyes, has been steadily increasing—globally, in both high- and low-income areas—over the past 50 years, paralleling the decline in public health [39–46] and the rise in EOCRC [44, 47–55].

Allura Red AC, designed to enhance the visual appeal of a wide range of food products, has become ubiquitous in modern diets, from candies and sodas to cereals and baked goods. Over 50% of processed food consumed in the USA now contains one or more of nine FDA-approved synthetic dyes [56], with Allura Red AC being the most commonly used in the USA, Canada, and many parts of the western world [57, 58]. Despite its global widespread use, concerns persist about the potential impact on human health, particularly as it is often used to market toward children, whose long-term exposure effects remain largely unknown. Since the 1970s, the FDA has witnessed a 3-fold increase in the certification of synthetic food dyes. Correspondingly, the amounts of Allura Red AC have surged by approximately 25-fold during this period [59]. Agencies like the Joint FAO/WHO Expert Committee on Food Additives (JECFA) and the EFSA established an Acceptable Daily Intake (ADI) for Allura Red at 7 mg/kg/day and No Observed Adverse Effect Level (NOAEL) of 695 mg/kg/day in 1981 [60–62]. However, this is based on outdated and sparse data from the 1960s and 1970s [62]. Although reevaluated in 1979, 1980, 1981, 1984, and 2016 [62], the ADI and NOAEL for Allura Red AC has not changed.

## How much Allura Red AC are people consuming?

Allura Red AC and other synthetic food dyes are widespread in our food supply, with a significant portion ending up in food and beverages [63]. Research by Bradman *et al.* [64] found that children, especially ages 5–9, have the highest exposure to Allura Red AC, primarily from juice drinks, soft drinks, icings, and ice cream cones. Lower-income, less-educated, and African American individuals tend to have higher intake [64]. Additionally, a study by Doell *et al.* [63] showed that over 90% of individuals consume foods containing common synthetic dyes, with Allura Red AC being the most consumed, particularly in soft and juice drinks targeted at children. Indeed, Allura Red AC has emerged as the most consumed dye across all age groups, notably prevalent in soft and juice drinks targeted toward children.

Contrary to these concerns, Bastaki *et al.* [65], representing the industry that employs synthetic food dyes, concluded from data obtained from NHANES 2009–2012 that current food color usage practices in the USA are safe and do not lead to excessive exposure to the population. As representatives of the industry that have a vested interest in selling synthetic food dyes [authors are from the International Association of Color Manufacturers (IACM), Coloron, The Coca-Cola Company, and Exponent], it is important to carry out additional scientific studies given the impact of Allura Red AC on inflammation and the microbiome, as described in the following sections.

Stevens *et al.* [59] showed that synthetic food dyes are widely used to color foods and beverages. Since the 1970s, the amount of synthetic food dyes that the FDA has certified has tripled. For Allura Red AC, this surge is 25-fold during this same period [45, 59]. They also showed that the average consumption of Allura Red AC in 1977 was approximately 9 mg/day (0.13 mg/kg/day). Overall, while actual levels consumed are relatively consistently below the ADI for most of the population [45, 59, 66], it should be noted that the ADI was set in 1981 and based on antiquated and sometimes unpublished scientific data; without considering the age of exposure [61]. The lack of rigorous, independent, and more current data necessitates further investigation into the sociodemographic determinants and toxicology of Allura Red AC and all synthetic food dyes. It is increasingly recognized that certain individuals and populations, such as children and minorities, are likely to experience heightened exposure and/or sensitivity due to unknown factors or genetic makeup. It is also possible that children and minorities experience chronic exposure to resulting chronic diseases, such as cancer. Thus, there is a growing consensus that children could easily surpass recommended intake levels solely through beverage consumption, highlighting the need for continued research into the impact of synthetic food dye’s mechanisms in carcinogenesis; especially in children. Indeed, it is also important to affect change through parallel policy and awareness campaigns.

## Allura Red AC interacts with key mediators of colorectal carcinogenesis

The following sections outline the intricate relationship between inflammation, the microbiome, and DNA damage in the context of Allura Red AC consumption (Graphical Abstract). Allura Red AC represents a xenobiotic that can potentially activate the immune system, fueling inflammation in the colon. The immune response triggered by Allura Red AC may involve both innate and adaptive immune mechanisms, leading to persistent low-grade inflammation. Furthermore, understanding the impact of Allura Red AC on the microbiome is crucial, as its metabolites may contribute to DNA damage and inflammation, potentially influencing colorectal health. Additionally, investigations into the genotoxic properties of Allura Red AC underscore the importance of exploring its potential role in promoting colorectal cancer, with findings indicating a variable impact on DNA integrity across preclinical models and a need for further research to translate these findings into human trials.

## Inflammation

Food consumption represents a significant source of xenobiotics that challenge the immune system [67, 68]. As a xenobiotic [1, 69, 70] Allura Red AC can activate the immune system and fuel inflammation in the colon [71]. When the body encounters a xenobiotic (such as Allura Red AC), it may trigger both innate and adaptive immune responses [72, 73]. The innate immune system may recognize the synthetic food dye as a foreign substance (xenobiotic), initiating a nonspecific immune response. It is reasonable that immune cells, such as macrophages and dendritic cells, may engulf the dye particles through a process called phagocytosis. Upon engulfment, these immune cells release cytokines, reactive oxygen species, and other signaling molecules that promote persistent and recurring low-grade inflammation. Simultaneously, the adaptive immune system can become involved. Antigen-presenting cells, such as dendritic cells, can process and present fragments of the synthetic food dye, called antigens, to T cells, which stimulates differentiation into effector T cells. Effector T cells, as well as innate cells (e.g. macrophages) release cytokines, reactive oxygen and nitrogen species, and other factors both damaging to the surrounding tissue and perpetuating inflammation.

The impact of Allura Red AC on the immune system remains mostly speculative yet testable. Understanding the impact of Allura Red AC on low-grade colon and rectal inflammation is of particular significance, as this is the site of EOCRCs [29, 74] and a western dietary pattern increases the risk of Colorectal Cancer (CRC) at this anatomic site [75, 76]. With this understanding, we hypothesized that Allura Red AC—acting as a xenobiotic—can cause low-grade inflammation in the distal colon and rectum of mice consuming it. Consistent with this hypothesis, we found that Allura Red AC damages DNA both *in vitro* and *in vivo* and that consumption of Allura Red AC (7 mg/kg/day) for 10 months leads to dysbiosis and low-grade colonic inflammation in the distal colon. There was also an increase in systemic Interleukin-6 (IL-6) concentrations, and inducible nitric oxide synthase (iNOS) in the colon [71]. This supports the notion that Allura Red AC induces a subtle and low-grade inflammatory response specific to the colon and rectum. Over years of consistent consumption, this may become dangerous, and contribute to the development of CRC, especially in the distal colon and rectum.

Others have shown that Allura Red AC interacts with other molecular players involved in inflammation. Allura Red AC augments the *in vitro* synthesis of leukotriene B4 and F2-isoprostanes from blood neutrophils [71]. It also inhibits the esterase activity of carbonic anhydrase II *in vitro* [77]. Interestingly, carbonic anhydrases are drug targets for a variety of diseases and their inhibitors are in clinical use as drugs for the management of glaucoma, epilepsy, obesity, and tumors [78]. As interesting, Ibuprofen—a common anti-inflammatory—is an inhibitor of carbonic anhydrase II [79]. The role of carbonic anhydrases in inflammation is further supported by the understanding that inhibitors of this target suppress the activation of macrophages [80]. Finally, inflammation can be prevented by a C1-esterase inhibitor [81].

Male Wister albino rats fed the human equivalent of 7 mg/kg/day Allura Red AC for 4 weeks causes an increase in pro-carcinogenic markers, including DNA damage, and systemic aspartate aminotransferase (AST), alanine aminotransferase (ALT), creatinine, malondialdehyde (MDA), nitric oxide (NO), and cyclooxygenase-2 (Cox-2) [82]. As well, there was lower total antioxidant capacity and histopathological and physiological aberrations in the liver and kidney of these rats [82]. With regard to the colon, in addition to our recent findings [71], He *et al.* [83] showed that interleukin 23 (IL-23) is a pivotal molecule at the crossroads of Allura Red AC and colon inflammation. They showed that Allura Red AC triggers Inflammatory Bowel Disease (IBD)-like colitis in mice over-expressing IL-23, leading to an increased generation of activated CD4^+^ T cells that express interferon-γ (IFN-γ). Importantly, colitis induction was dependent on the commensal microbiota promoting the azo reduction of Allura Red AC and generation of a metabolite, 1-amino-2-naphthol-6-sulfonate sodium salt. In 2022, Chen *et al.* [84] showed that Allura Red AC is an environmental risk factor for colitis development in mice with increased expression of interleukin IL-23. This immune response is mediated by IFN-γ-producing CD4^+^ cells [84]. In 2022, Kwon *et al.* [85] showed that chronic exposure to a relevant dose of Allura Red AC exacerbates colitis in mice, with intermittent exposure over 12 weeks showing no impact on colitis susceptibility. Early-life exposure primes mice for heightened susceptibility to colitis, while chronic exposure induces mild colitis [85].

Mouse colitis induced by Allura Red AC increases colonic serotonin levels and impairs epithelial barrier function via myosin light chain kinase. This effect is absent in mice lacking tryptophan hydroxylase 1, the enzyme for serotonin synthesis. Transferring altered gut microbiota from exposed mice worsens colitis severity in germ-free mice, and chronic exposure raises colonic serotonin levels. Though its impact on humans is uncertain, these findings suggest long-term exposure promotes colitis in mice via microbiota-dependent and -independent pathways involving serotonin. Limited but robust studies indicate Allura Red AC interacts with inflammatory and immune systems, necessitating further exploration. Among the observed consequences, colitis seems to be a notable outcome in a background of dysfunctional pathways such as that of those outlined in Table 1. Although the potential link to cancer in susceptible individuals remains a matter for future scientific investigation, it is clear that Allura Red AC interacts with the inflammatory machinery.

**Table 1.** Key inflammatory molecules that Allura Red AC interacts with.

Molecule	How it interacts	Reference
IL-6	Mice consuming Allura Red AC have high systemic IL-6 levels.	[71]
iNOS	Mice consuming Allura Red AC have high colon iNOS levels.	[71]
Leukotriene B4 (LTB4)	Allura Red AC augments synthesis of LTB4 *in vitro*.	[77]
F2-isoprostanes	Allura Red AC augments the synthesis of F2-isoprostanes *in vitro*.	[77]
Carbonic anhydrase II	Allura Red AC inhibits the esterase activity of carbonic anhydrase II *in vitro*.	[77]
Aspartate aminotransferase (AST)	Male rats consuming Allura Red AC have high systemic AST levels.	[82]
Alanine aminotransferase (ALT)	Male rats consuming Allura Red AC have high systemic ASP levels.	[82]
Creatinine	Male rats consuming Allura Red AC have high systemic creatine levels.	[82]
Malondialdehyde	Male rats consuming Allura Red AC have high systemic malondialdehyde levels.	[82]
Cyclooxygenase-2 (Cox-2)	Male rats consuming Allura Red AC have high systemic Cox-2 levels.	[82]
Antioxidant capacity	Male rats consuming Allura Red AC have lower systemic total antioxidant capacity.	[82]
IL-23	Allura Red AC triggers an IBD-like colitis in mice over-expressing IL-23.	[83, 84]
Serotonin (5-HT)	Chronic Allura Red AC exposure induced mouse colitis through colonic serotonin (5-HT).	[85]
Myosin light chain kinase (MLCK)	Chronic Allura Red AC exposure induced impairment of the epithelial barrier function mediated by MLCK.	[85]

## Microbiome

The intestinal microbiota plays a pivotal role in human health and disease by maintaining intestinal balance through intricate mechanisms. Substantial research highlights distinct differences in the gut microbiota between individuals with gastrointestinal tumors and healthy counterparts [86–88]. The dysregulation of gut microbiota, coupled with metabolites produced by gut bacteria and associated signaling pathways, offers insights into the mechanisms driving the occurrence and progression of gastrointestinal tumors. It also offers insight into the interactive role of the gut microbiome and the foods we eat [89, 90].

**Figure 1:**
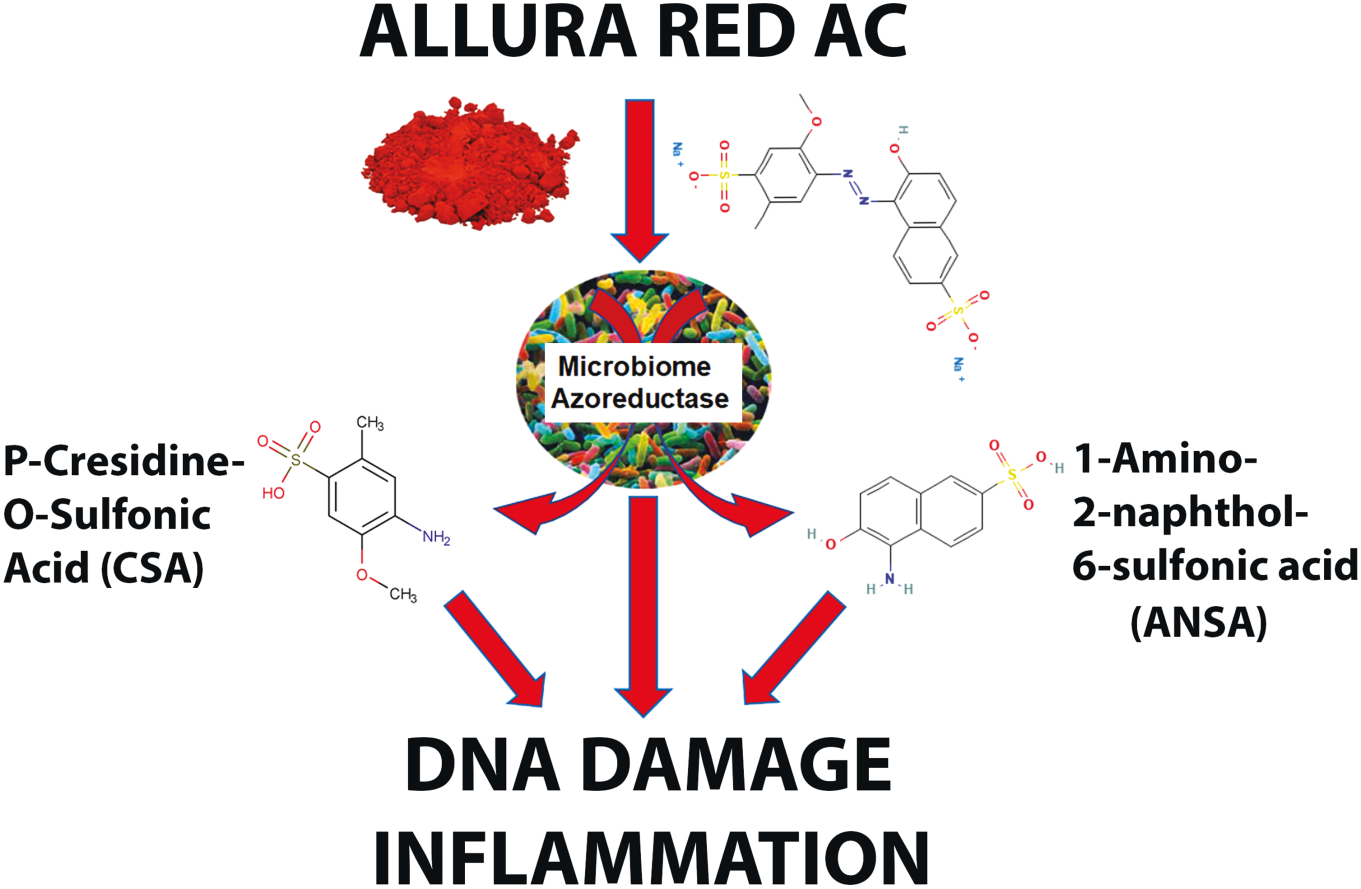
Allura Red AC is a xenobiotic and is metabolized in the gut microbiome, where azoreductases cleave the azo bonds of the dye into cresidine-4-sulfonic acid (CSA) and 1-amino-2-naphthol-6-sulfonic acid (ANSA). Evidence suggests these metabolites are DNA-reactive and activate the inflammatory machinery.

Understanding the effects of Allura Red AC on the microbiome is crucial, as it is metabolized by intestinal bacteria through “azo reduction.” Indeed, the microbiome is a critical component of the genesis of CRC [91, 92]. Despite its impact on short and long-term health in susceptible people, and the wide range of people consuming Allura Red AC, the interaction of this synthetic molecule with the microbiome has been inadequately studied, with few published studies investigating this interaction [71, 93]. Allura Red is a sulfonated mono azo dye; and as such, is metabolized by some intestinal bacteria [83, 94, 95] (Table 2). This xenobiotic is metabolized in the gut microbiome, where azoreductases cleave the azo bonds of the dye [93, 94]. The two known metabolites of Allura Red AC resulting from this cleavage are cresidine-4-sulfonic acid and 1-amino-2-naphthol-6-sulfonic acid; both of which have the potential to form DNA adducts, and have pro-inflammatory properties [1, 60, 82, 96] (Fig. 1). It was recently shown that Allura Red AC is a potent inhibitor of the key intestinal transporter OATP2B1, indicating its potential to impede drug absorption as well, depending on the makeup of the gut microbiome [97].

**Table 2.** Metabolism of Allura Red AC by the microbiome.

Bacteria				
Yes, azo reduction				
*Fusobacterium* [93]	*Bacteroides* [93]	*Bifidobacterium* [93]	*Citrobacter* [93]	*B. ovatus* [83]
*Firmicutes* [83]	*Bacteroidetes* [83]	*Actinobacteria* [83]	*Bifidobacterium bifidum* [83]	*Collinsella aerofaciens* [83]
*E. faecalis* [83]	*Enterocloster* [98]	*Veillonella* [98]	*Clostridium pacaense* [98]	*P. vulgatus* [98]
*Bacteroides fragilis* [83]	*B. vulgatus* [83]	*Odoribacter splanchnicus* [83]	*Clostridium* [98]	*Hungatella* [98]
*Dielma* [98]	*Eggerthella* [98]	*Odoribacter* [98]	*Phocaeicola* [98]	*Clostridium symbiosum* [98]
*Enterocloster bolteae* [98]	*Enterocloster lavalensis* [98]	*Hungatella effluvi* [98]	*Hungatella hathewayi* [98]	*V. atypica* [98]
*Veillonella (V.) tobetsuensis* [98]	*V. nakazawae* [98]	*V. dispar* [98]	*Dielma fastidiosa* [98]	*Clostridium innocuum* [98]
*Barnesiella intestinihominis* [98]	*O. splanchnicus* [98]	*Eggerthella lenta* [98]	*V. parvula* [98]	
No, azo reduction				
*Acidamiococcus* [93]	*Peptostreptococcus* [93]	*E. coli* [83, 98]	*Ruminococcus torques* [98]	*Coprococcus catus* [98]
*Anaerostipes hadrus* [98]	*Barnesiella intestinihominis* [98]	*Bacteroides kribbi* [98]	*Clostridium symbiosum* [98]	*Enterocloster bolteae* [98]

Despite the significant role of the microbiome in health; and the wide range of people consuming Allura Red AC, it is striking that so few scientists have studied the interaction of this xenobiotic with the microbiome. Many human gut bacterial species can reduce azo food dyes [98]. In 1978, Chung *et al.* showed high metabolism by *Fusobacterium*; modest metabolism by *Bacteroides*, *Bifidobacterium*, and *Citrobacter*; and that Allura Red AC is not metabolized by *Acidamiococcus* and *Peptostreptococcus* [93].

In 2022, He *et al.* [83] showed that Allura Red AC-induced colitis is dependent on *bacteroides*; and can be metabolized by *Bacteroides ovatus* (*B. ovatus)* and *Enterococcus faecalis (E. faecalis)*; but not *Escherichia coli* [83]. Particularly active in azo reduction in this study were bacteria from the three major phyla found in the gut: *Bacteroidetes*, *Firmicutes*, and *Actinobacteria*; and six bacterial species, including *Bifidobacterium bifidum*, *Collinsella aerofaciens*, *Bacteroides fragilis*, *B. ovatus*, *Bacteroides vulgatus (B. vulgatus)*, and *Odoribacter splanchnicus* [83]. In 2023, Elder *et al.* [98] tested the capacities of bacterial isolates from the human gut microbiome to reduce common azo food dyes *in vitro*. They showed taxa exhibiting high azoreductase activity in this survey belonged to the genera *Clostridium*, *Hungatella*, *Enterocloster*, *Veillonella*, *Dielma*, *Eggerthella*, *Odoribacter*, and *Phocaeicola*, which together represent a large fraction of the common bacterial commensals found in the human microbiome [98]. Of note, across studies, *E. coli* is a poor azo-dye reducer [83, 98]; and the species, *Odoribacter. splanchnicus(O. spanchnicus*), and *Phocaeicola vulgatus (P. vulgatus)*, appear to reduce Allura Red AC efficiently across studies [97, 98].

Overall, azoreductase activity is widespread among members of the gut microbiome, and the lack of a clear understanding of the potential toxic consequences of this azoreductive activity gives further impetus to the reevaluation of the prevalent use of azo synthetic food dyes as coloring agents in foods. Given the significance of the gut microbiome to gut health, that Allura Red AC is metabolized by the gut microbiome, and that Allura Red AC is consumed by a diverse and wide swath of people, understanding this interaction is of high significance to understanding colon and rectal health associated with synthetic food dyes.

## DNA damage

DNA damage is associated with oncogene activation and suppressor gene deactivation [99]; and can play a pivotal role in the development of CRC [100, 101], and the presence of synthetic food dyes such as Allura Red AC may contribute to this process. In this context, the potential genotoxic and mutagenic properties of Allura Red AC raise concerns about its impact on DNA integrity and the potential for promoting CRC. Also, Allura Red AC has been found to be contaminated with benzidine, 4-nitro-*p*-cresidine, and *p*-cresidine—which damage DNA [1, 102–104].

Several groups—including ourselves—have investigated the impact of Allura Red AC on DNA damage *in vitro* and *in vivo* (Supplementary Table S1). As such, there have been both negative and positive findings. In 2015, a group led by the IACM, Colorcon, and The Coca-Cola Company published a paper showing Allura Red AC did not induce DNA damage in the liver, stomach, and colon in adult male Harlan Sprague Dawley: Institute for Cancer Research (Hsd:ICR) (CD-1) mice consuming ADI levels of Allura Red for 45 h [105]. In the same year, a study published out of Japan with a single author showed no DNA damage, clastogenicity, or mutagenicity by Allura Red after 48 h in adult male CD2F1 mice [106]. In male Friend Virus B-type (FVB) mice given intraperitoneal (IP) injection of Allura Red, there was no detected DNA damage after 46 h [107].

In contrast, we [71] and others have found a positive association between Allura Red AC and DNA damage. In 2013, Jabeen *et al.* showed Allura Red AC exhibits genotoxic effects directly in *Saccharomyces cerevisiae* [108]. We have shown that Allura Red AC causes DNA damage *in vitro* and *in vivo* [71]; results supported by independent groups [95, 109, 110]. In 2001, 2002, and 2010, there were three studies published showing that Allura Red AC causes DNA damage to the colon after 3 h of consuming one dose of 10 mg/kg (human equivalent to 1 mg/kg/day) in adult male CD-1 (ICR) and ddY mice [109, 110]; but interestingly, not in adult male Fischer (F344) rats [95]. Our study showed Allura Red AC given at a human equivalent dose of 7 mg/kg (ADI) and 14 mg/kg (2× ADI) for 6 h, 24 h, or 1 week causes DNA damage in cells collected from the colons of female A/J mice [71]. Together, the preclinical animal models tested have led to variable results leading us to conclude that there is a potential impact of Allura Red AC on DNA damage in the colon. It is important to understand the epidemiology linking the consumption of Allura Red AC to DNA damage in the human colon. The sex and age differential responses will also be a critical avenue of experimentation to explore. Interestingly, all negative studies used males implicating a potential sex difference.

## Conclusions

First introduced and approved for use in food in the USA in 1971, Allura Red AC has become one of the most extensively used food colorants globally. While the scientific evidence is sparse, there is increasing independent evidence that this xenobiotic interacts with the mechanisms that control CRC (inflammation, DNA damage, and microbiome perturbations). Despite this, it remains in our food and consumption has increased drastically over the last 50 years due to the rise in the production and consumption of ultra-processed foods. Regulation of this food additive has been difficult. This will take effort on several fronts, including government intervention through regulatory bodies, such as the FDA in the USA and the EFSA in Europe, to continue to comprehensively evaluate and assess food dyes like Allura Red AC. In the USA, a key law involves a 1958 clause introduced into the Food, Drug and Cosmetic Act of 1938. This Delaney Clause stipulates “no cancer-causing agent, as demonstrated in humans or animals, shall be deliberately added to, or found as a contaminant in food” [111]. Thus, results consistent with the hypotheses suggested in this review will serve appropriate oversight agencies (e.g. FDA) in their assessment of Allura Red AC on health and the consideration of enacting the Delaney Clause to prohibit the further use of Allura Red in the USA.

As of April 2024, Allura Red AC is not banned outright in any country, but its usage is regulated in several places. For instance, in the EU and Canada, Allura Red AC is permitted for use in food products, but it requires special labeling if it is used. In the EU, the label must include the specific name of the color, i.e. “Allura Red AC,” followed by the E number (E129). In Norway and Finland, strict regulations have been implemented regarding food additives, including Allura Red AC. The USA has remained less conservative regarding food labeling. There, Allura Red AC and eight other synthetic food dyes are allowed for use in food and beverages. However, most products require specific labeling if they contain artificial colors like Allura Red AC. In a significant development in October 2023, California made history by becoming the first US state to ban products containing specific food additives, notably Red Dye No. 3 (Erythrosine). This regulation, slated to come into force in 2027, provides manufacturers with a grace period to remove the prohibited ingredients. It remains to be seen whether artificial dyes will be directly replaced or if some food producers will explore “dye-free” alternatives. Regardless, this legislation is expected to have a substantial impact on the broader US market, given the anticipated widespread production of reformulated products.

As research continues to explore the health implications of synthetic additives like Allura Red AC, integrative healthcare providers can play a role in promoting dietary changes that support overall well-being. Emphasizing the reduction of processed foods and unhealthy fats can help lower exposure to synthetic dyes, while encouraging the consumption of nutrient-rich alternatives, such as fruits and vegetables, is essential. Promoting anti-inflammatory diets can be achieved by reintroducing people to the natural flavors of whole foods and utilizing innovative techniques such as taste retraining. Restructuring incentives, such as redirecting subsidies toward nutrient-dense, plant-based foods, can make healthy options more accessible and affordable, thereby reducing both organismal and environmental inflammation. Supporting local agricultural initiatives like community-supported agriculture programs and farmers’ markets not only enhances food security but also encourages sustainable farming practices and increases access to fresh, nutritious foods. Additionally, promoting home gardening empowers individuals to grow their own anti-inflammatory foods, fostering a deeper connection with nature while improving access to fresh produce and encouraging physical activity. The recent regulatory actions against red dyes in California and the EU highlight growing concerns over synthetic additives, leading to shifts within the food industry. Addressing the challenge of Allura Red AC in our food supply requires a multifaceted approach. Solutions should range from scientific investigation to individual behavior changes, policy interventions, and collaborative efforts across various sectors, including industry and government.

## Supplementary Material

bgae057_suppl_Supplementary_Table_S1

## Data Availability

No new data were generated or analyzed in support of this review.

## References

[1] Kobylewski S , Jacobson MF. Toxicology of food dyes. Int J Occup Environ Health 2012;18:220–46. 23026007 10.1179/1077352512Z.00000000034

[2] Bakthavachalu P , Kannan SM, Qoronfleh MW. Food color and autism: a meta-analysis. Adv Neurobiol 2020;24:481–504. 32006369 10.1007/978-3-030-30402-7_15

[3] Dinç E , Ünal N, Ertekin ZC. Three-way analysis-based pH-UV-Vis spectroscopy for quantifying allura red in an energy drink and determining colorant’s pKa. J Food Drug Anal 2021;29:76–86. 35696222 10.38212/2224-6614.1275 PMC9261852

[4] Olas B , Białecki J, Urbańska K et al. The effects of natural and synthetic blue dyes on human health: a review of current knowledge and therapeutic perspectives. Adv Nutr 2021;12:2301–11. 34245145 10.1093/advances/nmab081 PMC8634323

[5] Vojdani A , Vojdani C. Immune reactivity to food coloring. Altern Ther Health Med 2015;21:52–62. 25599186

[6] Miller FW. The increasing prevalence of autoimmunity and autoimmune diseases: an urgent call to action for improved understanding, diagnosis, treatment, and prevention. Curr Opin Immunol 2023;80:102266. 36446151 10.1016/j.coi.2022.102266 PMC9918670

[7] McCann D , Barrett A, Cooper A et al. Food additives and hyperactive behaviour in 3-year-old and 8/9-year-old children in the community: a randomised, double-blinded, placebo-controlled trial. Lancet 2007;370:1560–7. 17825405 10.1016/S0140-6736(07)61306-3

[8] Al-Shihabi F , Moore A, Chowdhury TA. Diabetes and climate change. Diabet Med 2023;40:11. 10.1111/dme.14971 36209378

[9] El-Kassas M , Alswat K, Tharwat M et al. Steatotic liver disease as a new nomenclature for NAFLD from the perspectives of the MENA region: one size fits all this time. J Hepatol 2023;80:e66–8. 37619930 10.1016/j.jhep.2023.08.012

[10] Kokkorakis M , Boutari C, Katsiki N et al. From non-alcoholic fatty liver disease (NAFLD) to steatotic liver disease (SLD): an ongoing journey towards refining the terminology for this prevalent metabolic condition and unmet clinical need. Metabolism 2023;147:155664. 37517792 10.1016/j.metabol.2023.155664

[11] Rinella ME , Lazarus JV, Ratziu V et al.; NAFLD Nomenclature consensus group. A multi-society Delphi consensus statement on new fatty liver disease nomenclature. Ann Hepatol 2023;29:101133. 37364816 10.1016/j.aohep.2023.101133

[12] Miller KD , Fidler-Benaoudia M, Keegan TH et al. Cancer statistics for adolescents and young adults, 2020. CA Cancer J Clin 2020;70:443–59. 32940362 10.3322/caac.21637

[13] Siegel RL , Jakubowski CD, Fedewa SA et al. Colorectal cancer in the young: epidemiology, prevention, management. Am Soc Clin Oncol Educ Book 2020;40:1–14. 10.1200/EDBK_279901 32315236

[14] Remington PL , Brownson RC; Centers for Disease Control and Prevention (CDC). Fifty years of progress in chronic disease epidemiology and control. MMWR Suppl 2011;60:70–7. 21976169

[15] Miller MD , Steinmaus C, Golub MS et al. Potential impacts of synthetic food dyes on activity and attention in children: a review of the human and animal evidence. Environ Health 2022;21:45. 35484553 10.1186/s12940-022-00849-9 PMC9052604

[16] Ribe E , Cezard GI, Marshall A et al. Younger but sicker? Cohort trends in disease accumulation among middle-aged and older adults in Scotland using health-linked data from the Scottish Longitudinal Study. Eur J Public Health 2024;34:696–703. 38604658 10.1093/eurpub/ckae062 PMC11293808

[17] Bishop NJ , Haas SA, Quiñones AR. Cohort trends in the burden of multiple chronic conditions among aging U.S. adults. J Gerontol B Psychol Sci Soc Sci 2022;77:1867–79. 35642746 10.1093/geronb/gbac070 PMC9535783

[18] Nigg JT , Lewis K, Edinger T et al. Meta-analysis of attention-deficit/hyperactivity disorder or attention-deficit/hyperactivity disorder symptoms, restriction diet, and synthetic food color additives. J Am Acad Child Adolesc Psychiatry 2012;51:86–97.e8. 22176942 10.1016/j.jaac.2011.10.015 PMC4321798

[19] Hebert JR. Chapter 2. History of nutrition and inflammation. In: Hebert JR, Hofseth LJ (eds.), Diet, Inflammation, and Health. London: Academi Press/Elsevier Science, 2022.

[20] Hebert JR. Chapter 16. What constitutes an anti-inflammatory diet? How does this contrast with a pro-inflammatory diet? In: Hebert JR, Hofseth LJ (eds.), *Diet, Inflammation, and Health*. London: Academic Press/Elsevier Science, 2022.

[21] Novais C , Molina AK, Abreu RMV et al. Natural food colorants and preservatives: a review, a demand, and a challenge. J Agric Food Chem 2022;70:2789–805. 35201759 10.1021/acs.jafc.1c07533 PMC9776543

[22] Nakamura K et al. Results of the product examination of coal-tar dyes (including dye aluminum lakes) from April in 1978 till March in 1979 (on the product examination of coal-tar dyes XVIII). Eisei Shikenjo Hokoku 1979;97:173–4. 543954

[23] Barrows JN , Lipman AL, Bailey CJ. Color Additives History. Online: FDA; 2023.

[24] Krishan M , Navarro L, Beck B et al. A regulatory relic: after 60 years of research on cancer risk, the Delaney Clause continues to keep us in the past. Toxicol Appl Pharmacol 2021;433:115779. 34737146 10.1016/j.taap.2021.115779

[25] Lehto S , Buchweitz M, Klimm A et al. Comparison of food colour regulations in the EU and the US: a review of current provisions. Food Addit Contam Part A Chem Anal Control Expo Risk Assess 2017;34:335–55. 28004607 10.1080/19440049.2016.1274431

[26] FDA. Color Additive Status List. Online: FDA, 2023.

[27] FDA. Summary of Color Additives for Use in the United States in Foods, Drugs, Cosmetics, and Medical Devices. Online: FDA, 2023.

[28] Joint FAO/WHO Expert Committee on Food Additives. Evaluation of certain food additives. Twenty-third report of the Joint FAO; WHO Expert Committee on Food Additives. 1980.

[29] Hofseth LJ , Hebert JR, Chanda A et al. Early-onset colorectal cancer: initial clues and current views. Nat Rev Gastroenterol Hepatol 2020;17:352–64. 32086499 10.1038/s41575-019-0253-4 PMC10711686

[30] Murray CJ , Atkinson C, Bhalla K et al.; U.S. Burden of Disease Collaborators. The state of US health, 1990–2010: burden of diseases, injuries, and risk factors. JAMA 2013;310:591–608. 23842577 10.1001/jama.2013.13805 PMC5436627

[31] Noorafshan A , Hashemi M, Karbalay-Doust S et al. High dose Allura Red, rather than the ADI dose, induces structural and behavioral changes in the medial prefrontal cortex of rats and taurine can protect it. Acta Histochem 2018;120:586–94. 30031538 10.1016/j.acthis.2018.07.004

[32] Al-Shabib NA , Khan JM, Malik A et al. Allura red rapidly induces amyloid-like fibril formation in hen egg white lysozyme at physiological pH. Int J Biol Macromol 2019;127:297–305. 30654033 10.1016/j.ijbiomac.2019.01.049

[33] Lv JL , Wei Y-F, Sun J-N et al. Ultra-processed food consumption and metabolic disease risk: an umbrella review of systematic reviews with meta-analyses of observational studies. Front Nutr 2024;11:1306310. 38356860 10.3389/fnut.2024.1306310 PMC10864658

[34] Isaksen IM , Dankel SN. Ultra-processed food consumption and cancer risk: a systematic review and meta-analysis. Clin Nutr 2023;42:919–28. 37087831 10.1016/j.clnu.2023.03.018

[35] Elizabeth L , Machado P, Zinöcker M et al. Ultra-processed foods and health outcomes: a narrative review. Nutrients 2020;12:1955. 32630022 10.3390/nu12071955 PMC7399967

[36] Lane MM , Gamage E, Du S et al. Ultra-processed food exposure and adverse health outcomes: umbrella review of epidemiological meta-analyses. BMJ 2024;384:e077310. 38418082 10.1136/bmj-2023-077310 PMC10899807

[37] Menichetti F , Leone A. Consumption of ultra-processed foods and health harm. Nutrients 2023;15:2945. 37447271 10.3390/nu15132945 PMC10346525

[38] Alves R , Lopes C, Perelman J. Healthy eating: a privilege for the better-off? Eur J Clin Nutr 2022;76:134–42. 33986488 10.1038/s41430-021-00926-1

[39] Martínez Steele E , Baraldi LG, Louzada MLC et al. Ultra-processed foods and added sugars in the US diet: evidence from a nationally representative cross-sectional study. BMJ Open 2016;6:e009892. 10.1136/bmjopen-2015-009892 PMC4785287 26962035

[40] Aguayo-Patron SV , Calderón de la Barca AM. Old fashioned vs. ultra-processed-based current diets: possible implication in the increased susceptibility to type 1 diabetes and celiac disease in childhood. Foods 2017;6:100. 29140275 10.3390/foods6110100 PMC5704144

[41] da Silva A , Felício MB, Caldas APS et al. Pro-inflammatory diet is associated with a high number of cardiovascular events and ultra-processed foods consumption in patients in secondary care. Public Health Nutr 2020;24:3331–40. 33148359 10.1017/S136898002000378X PMC10195276

[42] Kliemann N , Al Nahas A, Vamos EP et al. Ultra-processed foods and cancer risk: from global food systems to individual exposures and mechanisms. Br J Cancer 2022;127:14–20. 35236935 10.1038/s41416-022-01749-y PMC9276654

[43] Srour B , Kordahi MC, Bonazzi E et al. Ultra-processed foods and human health: from epidemiological evidence to mechanistic insights. Lancet Gastroenterol Hepatol 2022;7:1128–40. 35952706 10.1016/S2468-1253(22)00169-8

[44] Zhang Y , Giovannucci EL. Ultra-processed foods and health: a comprehensive review. Crit Rev Food Sci Nutr 2022;63:10836–48. 35658669 10.1080/10408398.2022.2084359

[45] Stevens LJ , Burgess JR, Stochelski MA et al. Amounts of artificial food colors in commonly consumed beverages and potential behavioral implications for consumption in children: revisited. Clin Pediatr (Phila) 2015;54:1228–30. 25876924 10.1177/0009922815581348

[46] Jung S , Kim JY, Park S. Eating patterns in Korean adults, 1998–2018: increased energy contribution of ultra-processed foods in main meals and snacks. Eur J Nutr 2024;63:279–89. 37999737 10.1007/s00394-023-03258-x PMC10799128

[47] Hang D et al. Ultra-processed food consumption and risk of colorectal cancer precursors: results from three prospective cohorts. J Natl Cancer Inst 2022;115:155–164. 10.1093/jnci/djac221 PMC9905956 36477589

[48] Nguyen LH , Cao Y, Hur J et al. The sulfur microbial diet is associated with increased risk of early-onset colorectal cancer precursors. Gastroenterology 2021;161:1423–32.e4. 34273347 10.1053/j.gastro.2021.07.008 PMC8545755

[49] Lima Oliveira M et al. A perspective review on diet quality, excess adiposity and chronic psychosocial stress and implications for early-onset colorectal cancer. J Nutr 2024;154:1069–79. 38453027 10.1016/j.tjnut.2024.03.002 PMC11007745

[50] Hua H , Jiang Q, Sun P et al. Risk factors for early-onset colorectal cancer: systematic review and meta-analysis. Front Oncol 2023;13:1132306. 37213277 10.3389/fonc.2023.1132306 PMC10196487

[51] Ugai T , Liu L, Tabung FK et al. Prognostic role of inflammatory diets in colorectal cancer overall and in strata of tumor-infiltrating lymphocyte levels. Clin Transl Med 2022;12:e1114. 36437503 10.1002/ctm2.1114 PMC9702366

[52] Yue Y , Hur J, Cao Y et al. Prospective evaluation of dietary and lifestyle pattern indices with risk of colorectal cancer in a cohort of younger women. Ann Oncol 2021;32:778–86. 33812017 10.1016/j.annonc.2021.03.200 PMC8862727

[53] Joh HK , Lee DH, Hur J et al. Simple sugar and sugar-sweetened beverage intake during adolescence and risk of colorectal cancer precursors: adolescent sugar intake and colorectal polyp. Gastroenterology 2021;161:128–42.e20. 33753105 10.1053/j.gastro.2021.03.028 PMC8238879

[54] Hu J et al. Sugar-sweetened beverage intake in adulthood and adolescence and risk of early-onset colorectal cancer among women. Gut 2021;70:2330–6. 33958435 10.1136/gutjnl-2020-323450 PMC8571123

[55] Chen H , Zheng X, Zong X et al. Metabolic syndrome, metabolic comorbid conditions and risk of early-onset colorectal cancer. Gut 2020;70:1147–54. 33037055 10.1136/gutjnl-2020-321661 PMC8032822

[56] Ha MS , Ha S-D, Choi S-H et al. Exposure assessment of synthetic colours approved in Korea. Food Addit Contam Part A Chem Anal Control Expo Risk Assess 2013;30:643–53. 23521141 10.1080/19440049.2013.768358

[57] Amchova P , Kotolova H, Ruda-Kucerova J. Health safety issues of synthetic food colorants. Regul Toxicol Pharmacol 2015;73:914–22. 26404013 10.1016/j.yrtph.2015.09.026

[58] Potera C. The artificial food dye blues. Environ Health Perspect 2010;118:A428. 20884387 10.1289/ehp.118-a428 PMC2957945

[59] Stevens LJ , Burgess JR, Stochelski MA et al. Amounts of artificial food colors in commonly consumed beverages and potential behavioral implications for consumption in children. Clin Pediatr (Phila) 2014;53:133–40. 24037921 10.1177/0009922813502849

[60] JECFA. Evaluation of certain food additives: eighty-second report of the Joint FAO/WHO Expert Committee on Food Additives. World Health Organization technical report series. 2016, 1–162. 4623237

[61] JECFA. Twenty-fifth report of the Joint FAO/WHO Expert Committee on Food Additives meeting held in Geneva from 23 March to 1 April 1981. World Health Organization, 1981.

[62] Ramos-Souza C , Bandoni DH, Bragotto APA et al. Risk assessment of azo dyes as food additives: revision and discussion of data gaps toward their improvement. Compr Rev Food Sci Food Saf 2022;22:380–407. 36374221 10.1111/1541-4337.13072

[63] Doell DL , Folmer DE, Lee HS et al. Exposure estimate for FD&C colour additives for the US population. Food Addit Contam Part A Chem Anal Control Expo Risk Assess 2016;33:782–97. 27092991 10.1080/19440049.2016.1179536 PMC4975380

[64] Bradman A , Castorina R, Thilakaratne R et al. Dietary exposure to united states food and drug administration-approved synthetic food colors in children, pregnant women, and women of childbearing age living in the United States. Int J Environ Res Public Health 2022;19:9661. 35955015 10.3390/ijerph19159661 PMC9368057

[65] Bastaki M , Farrell T, Bhusari S et al. Estimated daily intake and safety of FD&C food-colour additives in the US population. Food Addit Contam Part A Chem Anal Control Expo Risk Assess 2017;34:891–904. 28332449 10.1080/19440049.2017.1308018

[66] Stevens LJ , Burgess JR, Stochelski MA et al. Amounts of artificial food dyes and added sugars in foods and sweets commonly consumed by children. Clin Pediatr (Phila) 2015;54:309–21. 24764054 10.1177/0009922814530803

[67] Nogacka AM , Gómez-Martín M, Suárez A et al. Xenobiotics formed during food processing: their relation with the intestinal microbiota and colorectal cancer. Int J Mol Sci 2019;20:2051. 31027304 10.3390/ijms20082051 PMC6514608

[68] Ortiz P , Torres-Sánchez A, López-Moreno A et al. Impact of cumulative environmental and dietary xenobiotics on human microbiota: risk assessment for one health. J Xenobiot 2022;12:56–63. 35323221 10.3390/jox12010006 PMC8949313

[69] Kobylewski S , Jacobson ME. Food Dyes: A Rainbow of Risk. Center for Science in the Public Interest, 2010, 1–68.

[70] Kyriakides TR , Kim H-J, Zheng C et al. Foreign body response to synthetic polymer biomaterials and the role of adaptive immunity. Biomed Mater 2022;17. 10.1088/1748-605X/ac5574 PMC9159526 35168213

[71] Zhang Q , Chumanevich AA, Nguyen I et al. The synthetic food dye, Red 40, causes DNA damage, causes colonic inflammation, and impacts the microbiome in mice. Toxicol Rep 2023;11:221–32. 37719200 10.1016/j.toxrep.2023.08.006 PMC10502305

[72] Gowdy KM , Laskin DL. Resolution of inflammation in xenobiotic-induced mucosal injury and chronic disease. Toxicol Appl Pharmacol 2023;466:116455. 36907382 10.1016/j.taap.2023.116455

[73] Xie W , Tian Y. Xenobiotic receptor meets NF-kappaB, a collision in the small bowel. Cell Metab 2006;4:177–8. 16950133 10.1016/j.cmet.2006.08.004

[74] Siegel RL , Wagle NS, Cercek A et al. Colorectal cancer statistics, 2023. CA Cancer J Clin 2023;73:233–54. 36856579 10.3322/caac.21772

[75] Castello A et al. Low adherence to the Western and high adherence to the Mediterranean dietary patterns could prevent colorectal cancer. Eur J Nutr 2018;58:1495–505. 29582162 10.1007/s00394-018-1674-5

[76] Mehta RS , Song M, Nishihara R et al. Dietary patterns and risk of colorectal cancer: analysis by tumor location and molecular subtypes. Gastroenterology 2017;152:1944–53.e1. 28249812 10.1053/j.gastro.2017.02.015 PMC5447483

[77] Esmaeili S , Ashrafi-Kooshk MR, Khaledian K et al. Degradation products of the artificial azo dye, Allura red, inhibit esterase activity of carbonic anhydrase II: a basic in vitro study on the food safety of the colorant in terms of enzyme inhibition. Food Chem 2016;213:494–504. 27451209 10.1016/j.foodchem.2016.06.078

[78] Supuran CT. Multitargeting approaches involving carbonic anhydrase inhibitors: hybrid drugs against a variety of disorders. J Enzyme Inhib Med Chem 2021;36:1702–14. 34325588 10.1080/14756366.2021.1945049 PMC8330743

[79] Combs J , Andring J, McKenna R. Ibuprofen: a weak inhibitor of carbonic anhydrase II. Acta Crystallogr Sect F Struct Biol Commun 2022;78:395–402. 36322425 10.1107/S2053230X22009761 PMC9629514

[80] Berrino E , Carradori S, Angeli A et al. Dual carbonic anhydrase IX/XII inhibitors and carbon monoxide releasing molecules modulate LPS-mediated inflammation in mouse macrophages. Antioxidants (Basel) 2021;10:56. 33466457 10.3390/antiox10010056 PMC7824903

[81] Begieneman MP , Emmens RW, Rijvers L et al. Myocardial infarction induces atrial inflammation that can be prevented by C1-esterase inhibitor. J Clin Pathol 2016;69:1093–9. 27153875 10.1136/jclinpath-2016-203639

[82] Khayyat LI , Essawy AE, Sorour JM et al. Sunset Yellow and Allura Red modulate Bcl2 and COX2 expression levels and confer oxidative stress-mediated renal and hepatic toxicity in male rats. PeerJ 2018;6:e5689. 30280050 10.7717/peerj.5689 PMC6166620

[83] He Z , Chen L, Catalan-Dibene J et al. Food colorants metabolized by commensal bacteria promote colitis in mice with dysregulated expression of interleukin-23. Cell Metab 2021;33:1358–71.e5. 33989521 10.1016/j.cmet.2021.04.015 PMC8266754

[84] Chen L , He Z, Reis BS et al. IFN-γ(+) cytotoxic CD4(+) T lymphocytes are involved in the pathogenesis of colitis induced by IL-23 and the food colorant Red 40. Cell Mol Immunol 2022;19:777–90. 35468944 10.1038/s41423-022-00864-3 PMC9243055

[85] Kwon YH , Banskota S, Wang H et al. Chronic exposure to synthetic food colorant Allura Red AC promotes susceptibility to experimental colitis via intestinal serotonin in mice. Nat Commun 2022;13:7617. 36539404 10.1038/s41467-022-35309-y PMC9768151

[86] Tabowei G , Gaddipati GN, Mukhtar M et al. Microbiota dysbiosis a cause of colorectal cancer or not? A systematic review. Cureus 2022;14:e30893. 36465770 10.7759/cureus.30893 PMC9711892

[87] Artemev A , Naik S, Pougno A et al. The association of microbiome dysbiosis with colorectal cancer. Cureus 2022;14:e22156. 35174040 10.7759/cureus.22156 PMC8840808

[88] DeDecker L , Coppedge B, Avelar-Barragan J et al. Microbiome distinctions between the CRC carcinogenic pathways. Gut Microbes 2021;13:1854641. 33446008 10.1080/19490976.2020.1854641 PMC8288036

[89] Abu-Ghazaleh N , Chua WJ, Gopalan V. Intestinal microbiota and its association with colon cancer and red/processed meat consumption. J Gastroenterol Hepatol 2021;36:75–88. 32198788 10.1111/jgh.15042

[90] Appunni S , Rubens M, Ramamoorthy V et al. Emerging evidence on the effects of dietary factors on the gut microbiome in colorectal cancer. Front Nutr 2021;8:718389. 34708063 10.3389/fnut.2021.718389 PMC8542705

[91] He J , Li H, Jia J et al. Mechanisms by which the intestinal microbiota affects gastrointestinal tumours and therapeutic effects. Mol Biomed 2023;4:45. 38032415 10.1186/s43556-023-00157-9 PMC10689341

[92] Song M , Chan AT, Sun J. Influence of the gut microbiome, diet, and environment on risk of colorectal cancer. Gastroenterology 2020;158:322–40. 31586566 10.1053/j.gastro.2019.06.048 PMC6957737

[93] Chung KT , Fulk GE, Egan M. Reduction of azo dyes by intestinal anaerobes. Appl Environ Microbiol 1978;35:558–62. 25047 10.1128/aem.35.3.558-562.1978 PMC242879

[94] Feng J , Cerniglia CE, Chen H. Toxicological significance of azo dye metabolism by human intestinal microbiota. Front Biosci 2012;4:568–86. 10.2741/400 PMC5870118 22201895

[95] Shimada C , Kano K, Sasaki YF et al. Differential colon DNA damage induced by azo food additives between rats and mice. J Toxicol Sci 2010;35:547–54. 20686341 10.2131/jts.35.547

[96] Oplatowska-Stachowiak M , Elliott CT. Food colors: existing and emerging food safety concerns. Crit Rev Food Sci Nutr 2017;57:524–48. 25849411 10.1080/10408398.2014.889652

[97] Zou L , Spanogiannopoulos P, Pieper LM et al. Bacterial metabolism rescues the inhibition of intestinal drug absorption by food and drug additives. Proc Natl Acad Sci USA 2020;117:16009–18. 32571913 10.1073/pnas.1920483117 PMC7355017

[98] Elder R , Vancuren SJ, Botschner AJ et al. Metabolism of azo food dyes by bacterial members of the human gut microbiome. Anaerobe 2023;83:102783. 37769703 10.1016/j.anaerobe.2023.102783

[99] Cooks T , Pateras IS, Tarcic O et al. Mutant p53 prolongs NF-κB activation and promotes chronic inflammation and inflammation-associated colorectal cancer. Cancer Cell 2013;23:634–46. 23680148 10.1016/j.ccr.2013.03.022 PMC3657134

[100] Zou X , Koh GCC, Nanda AS et al.; Genomics England Research Consortium. A systematic CRISPR screen defines mutational mechanisms underpinning signatures caused by replication errors and endogenous DNA damage. Nat Cancer 2021;2:643–57. 34164627 10.1038/s43018-021-00200-0 PMC7611045

[101] Hofseth LJ , Khan MA, Ambrose M et al. The adaptive imbalance in base excision-repair enzymes generates microsatellite instability in chronic inflammation. J Clin Invest 2003;112:1887–94. 14679184 10.1172/JCI19757 PMC296999

[102] Delker DA , Yano BL, Gollapudi BB. Evaluation of cytotoxicity, cell proliferation, and genotoxicity induced by p-cresidine in hetero- and nullizygous transgenic p53 mice. Toxicol Sci 2000;55:361–9. 10828268 10.1093/toxsci/55.2.361

[103] Richfield-Fratz N , Baczynskyj WM, Miller GC et al. Isolation, characterization and determination of trace organic impurities in FD&C Red No. 40. J Chromatogr 1989;467:167–76. 2753932 10.1016/s0021-9673(01)93961-5

[104] Tsuji S , Umino Y, Amakura Y et al. [Preparation of HPLC test solutions for organic impurities in aluminum lakes of food red no. 40 (allura red AC) and food yellow no. 5 (sunset yellow FCF)]. Shokuhin Eiseigaku Zasshi 2001;42:379–84. 11875823 10.3358/shokueishi.42.379

[105] Bastaki M et al. Lack of genotoxicity in vivo for food color additive Allura Red AC. Food Chem Toxicol 2017;105:308–14. 28458012 10.1016/j.fct.2017.04.037

[106] Honma M. Evaluation of the in vivo genotoxicity of Allura Red AC (Food Red No. 40). Food Chem Toxicol 2015;84:270–5. 26364875 10.1016/j.fct.2015.09.007

[107] Abramsson-Zetterberg L , Ilback N-G. The synthetic food colouring agent Allura Red AC (E129) is not genotoxic in a flow cytometry–based micronucleus assay in vivo. Food Chem Toxicol 2013;59:86–9. 23748052 10.1016/j.fct.2013.05.047

[108] Jabeen HS , ur Rahman S, Mahmood S et al. Genotoxicity assessment of amaranth and allura red using *Saccharomyces cerevisiae*. Bull Environ Contam Toxicol 2013;90:22–6. 23132362 10.1007/s00128-012-0870-x

[109] Sasaki YF , Kawaguchi S, Kamaya A et al. The comet assay with 8 mouse organs: results with 39 currently used food additives. Mutat Res 2002;519:103–19. 12160896 10.1016/s1383-5718(02)00128-6

[110] Tsuda S , Murakami M, Matsusaka N et al. DNA damage induced by red food dyes orally administered to pregnant and male mice. Toxicol Sci 2001;61:92–9. 11294979 10.1093/toxsci/61.1.92

[111] Felter SP , Llewelyn C, Navarro L et al. How the 62-year old Delaney Clause continues to thwart science: case study of the flavor substance β-myrcene. Regul Toxicol Pharmacol 2020;115:104708. 32522581 10.1016/j.yrtph.2020.104708

