# Supplementary material for: Allura Red AC is a xenobiotic. Is it also a carcinogen?
Source: Carcinogenesis. 2024 Aug 12;45(10):711–20. doi: 10.1093/carcin/bgae057 (PMC11464682; doi:10.1093/carcin/bgae057)
Supplement: bgae057_suppl_Supplementary_Table_S1 [file bgae057_suppl_supplementary_table_s1.docx]

**Supplemental Table 1**.  Studies examining the impact of Allura Red AC on DNA.

| **Study** | **Dose of Allura Red AC and Methods** | **Outcome** |
| --- | --- | --- |
| *(102)* | **Dose**: 0 - 2000 mg/kg body weight administered oral gavage.  **Mice**: Adult male Hsd:ICR (CD-1) mice  **Endpoints**: Comet and micronucleus assay. | •No genotoxic activity in mice consuming Allura Red for 48 hours. |
| *(103)* | **Dose**: 0 - 2000 mg/kg body weight administered oral gavage.  **Mice**: Adult male CD2F1 mice.  **Endpoints**: Comet assay, micronucleus assay, and transgenic gene mutation assay. | •No DNA damage, clastogenicity, or mutagenicity occurred after 48 hours in mice. |
| *(104)* | **Dose**: 0 - 2000 mg/kg body weight solutions in PBS administered intraperitoneal.  **Mice:** Adult, male FVB mice.  **Endpoints:** Micronucleus assay. | •No detected DNA damage after 46 hours at any concentration. |
| *(1,99-101)* | **Dose**: N/A  **Mice**: N/A  **Endpoints**: Reversed-phase high-performance liquid chromatography. | •Allura Red AC found to have  benzidine, 4-nitro-p-cresidine, and p-cresidine – defined as Class 1 and 2B carcinogens by IARC; and damage DNA. |
| *(71, 105)* | **Dose**: 9.76 to 5,000 μg/mL.  **Cells**: Saccharomyces cerevisiae incubated at 28°C and 37°C.  **Endpoints**: Comet assay. | •At 28°C - no notable genotoxic effects.  •At 37°C - direct correlation between the concentration of Allura Red AC and comet tail length, with the minimum exposure as 1,250 μg/mL after 2 hours causing damage. |
| *(71)* | **Dose**:  *In Vitro:* 0 – 1500 uM.  *In Vivo*: Human equivalent of: 0, 7 mg/kg body weight per day (ADI), 14 mg/kg body weight per day (2x ADI) with and without a high fat diet for 9 months.  **Mice**: Adult, female A/J mice.  **Endpoints**: Comet assay, iNOS immunohistochemistry, microscopic inflammation, IL-6 plasma levels, p53 and APC mutations. | •DNA damage *in vitro (dose and time dependent)* and *in vivo.*  •*In vitro* minimum concentration to detect damage was 31.25 uM.  •Low-grade distal colon and rectum inflammation.  •Does not appear to change the general composition of the bacterial community.  •The introduction of the HFD with Red 40  decreases beneficial microbial communities and increases harmful microbial communities.  p53 is functionally mutated by Red 40. |
| *(90,106,107)* | **Dose**: 0 – 2000 mg/kg body weight.  **Mice**:  •Pregnant female and male CD-1 (ICR) mice.  •Adult male ddY mice.  •Adult Male Fischer (F344) rats  **Endpoints**: Comet assay. | •DNA damage in the colons of mice, but not rats.  •One dose of 10 mg/kg Allura Red (human equivalent to 1 mg/kg/d) caused DNA damage after 3 hours in mice. |
